# Interaction of reproductive tract infections with estrogen exposure on breast cancer risk and prognosis

**DOI:** 10.1186/s12905-023-02383-3

**Published:** 2023-05-08

**Authors:** YunQian Li, XingLi Gan, ZhuoZhi Liang, HengMing Ye, Ying Lin, Qiang Liu, XiaoMing Xie, LuYing Tang, ZeFang Ren

**Affiliations:** 1 School of Public Health, Sun Yat-Sen University, 74 Zhongshan 2Nd Rd, Guangzhou, 510080 China; 2 School of Basic Medical Science, Southern Medical University, Guangzhou, China; 3 Breast Tumor Center, Sun Yat-Senen Memorial Hospital, Sun Yat-Senen University, Guangzhou, China; 4 The First Affiliated Hospital, Sun Yat-Sen University, Guangzhou, China; 5 Sun Yat-Sen Memorial Hospital, Sun Yat-Sen University, Guangzhou, China; 6 The Cancer Center, Sun Yat-Sen University, Guangzhou, China; 7 The Third Affiliated Hospital, Sun Yat-Sen University, Guangzhou, 510630 China

**Keywords:** Breast cancer, Reproductive tract infections, Lifetime estrogen exposure, Risk, Prognosis

## Abstract

**Background:**

Reproductive tract infections influenced a series of inflammatory processes which involved in the development of breast cancer, while the processes were largely affected by estrogen. The present study aimed to explore the associations of breast cancer risk and prognosis with reproductive tract infections and the modification effects of estrogen exposure.

**Methods:**

We collected history of reproductive tract infections, menstruation and reproduction from 1003 cases and 1107 controls and a cohort of 4264 breast cancer patients during 2008–2018 in Guangzhou, China. We used logistic regression model to estimate the odds ratios (ORs) and 95% confidence intervals (CIs) for risk; Cox model was applied to estimate the hazard ratios (HRs) and 95% CIs for progression-free survival (PFS) and overall survival (OS).

**Results:**

It was found that previous reproductive tract infections were negatively associated with breast cancer risk (OR = 0.80, 95%CI, 0.65–0.98), particularly for patients with more menstrual cycles (OR = 0.74, 95%CI, 0.57–0.96). Patients with previous reproductive tract infections experienced better OS (HR = 0.61; 95% CI, 0.40–0.94) and PFS (HR = 0.84; 95% CI, 0.65–1.09). This protective effect on PFS was only found in patients with more menstrual cycles (HR = 0.52, 95% CI:0.34–0.79, *P*_interaction_ = 0.015).

**Conclusions:**

The findings suggested that reproductive tract infections may be protective for the initiation and development of breast cancer, particularly for women with a longer interval of lifetime estrogen exposure.

**Supplementary Information:**

The online version contains supplementary material available at 10.1186/s12905-023-02383-3.

## Background

The relationship between infections and cancer development has been of interest for many years [1]. Infections were found to be associated with cancer risks and were further confirmed to influence cancer prognosis [2–7]. As for female reproductive tract infections, the common gynecological diseases [8], were found to be associated with risk of uterine fibroids inversely [9, 10]. Further studies had shown that the infection influenced a series of inflammatory processes involving in breast cancer development [11], such as increasing leukocyte infiltration, over-expression of cytokines and chemokines, and activating nuclear factor-κB [12–14], which suggested that reproductive tract infections might affect the development of breast cancer. Limited studies had explored the relationship between this infection and the risk of breast cancer, and the association remained unclear [15–17]; the relationship between reproductive tract infection and breast cancer prognosis was unexplored.

In addition, it had been found that estrogen, an established breast cancer risk factor [18], exerted a dual-directional regulation effect on inflammatory pathways: at a high dose, it inhibited inflammation, while it would have no such effect or even an opposite effect at a low level [19]. This phenomenon suggested that estrogen exposure might modify the association of reproductive tract infections with risk and prognosis of breast cancer. In the present study, therefore, we investigated the associations of reproductive tract infections with breast cancer development and further explored the modification effect of estrogen exposure on the associations.

## Methods

### Study design

A case–control study and a cohort study were conducted to investigate the association of reproductive tract infections with and breast cancer risk and the effect of reproductive tract infections on the prognosis, respectively.

### Study population

#### Case–control study

Female patients with recent histologically diagnosed primary breast cancer between October 2008 and March 2012 in the First- and Second-Affiliated Hospitals and Sun Yat-sen University Cancer Center, Guangzhou, China, were consecutively included in this study. We excluded women who had metastasized breast cancer or reported a previous history of any cancers. Controls were recruited from women who attended a health check-up during the same period in the same hospitals and then frequency-matched to the cases on age (± 5 years) and. Women with major chronic disease or whose self- reported a history of cancer were excluded. A total of 1551 cases and 1605 cancer-free controls were interviewed using the same questionnaire by trained interviewers face-to-face [20]. We collected reproductive tract infections recordings from 1003 cases and 1107 controls (64.7% and 69.0% of those eligible, respectively).

#### Cohort study

The subjects were recruited in the GZBCS between October 2008 and January 2018, as described in previously study [21]. Patients pathologically confirmed with breast cancer were collected from the Cancer Center and the First and Second Affiliated Hospitals of Sun Yat-sen University in Guangzhou, China. A total of 5418 patients of primary breast cancer were eligible for this study. We excluded the patients who didn’t complete the follow-up (N = 339) and lacked the information of pre-diagnostic reproductive tract infections history (N = 815), yielding a sample of 4264 cases.

All subjects must have resided in the Guangzhou area for at least 5 years. This study was approved by the Ethics Committee of the School of Public Health at Sun Yat-sen University. Informed consent was obtained from each participant before the interviews.

### Data collection

Participants were interviewed by well trained investigators using a structured questionnaire in-person at baseline. The questionnaire collects the information about demographic characteristics (age, education, marital status, BMI, oral contraceptive use), family history of breast cancer (yes/no), menstrual history (age at menarche, age at menopause, and mean number of menstrual cycles per year), and reproductive history (total number of pregnancies, outcome of every pregnancy and duration of breastfeeding), and history of reproductive tract infections (including sexually transmitted diseases, endogenous infections, and iatrogenic infections) which occurred in the fallopian tubes, ovaries, uterus, vagina, cervix and vulva and caused by invasion of pathogens such as *Candida*, *Chlamydia trachomatis*, *Trichomonas vaginalis*, *Neisseria gonorrhoeae*, and human papillomavirus (as defined by WHO) [22]. The infections were diagnosed by doctors (mostly gynecologists) with the symptoms, such as vaginal discharge, genital itching/irritation, lower abdominal pain or fever, as well as pathogenic detection, or ultrasound exam and trial treatment when necessary.

We defined age at menopause as age at final menstrual period, after a 12-month of amenorrhea. Reproductive time was calculated by subtracting the age at menarche between the age at menopause. The duration of parity was defined as the sum for months of live or stillbirth. The duration of pregnancy was calculated as the sum for months of live or stillbirth, induced or spontaneous abortions. The breastfeeding duration was defined as the sum of months of breastfeeding during each birth. We calculated the number of menstrual cycles by subtracting 9 months for every pregnancy, breastfeeding duration for each live birth from the reproductive time, and then converted to years, after which it was multiplied by the reported average number of menstrual cycles per year [23, 24]. Lifetime estrogen variables were classified by tertiles.

The clinicopathological characteristics were obtained from the medical records in hospitals. Immunohistochemical tests was used to determine the status of estrogen receptor (ER), progesterone receptor (PR), and human epidermal growth factor receptor 2 (HER2) of breast cancer tissues by pathologists. Detailed definitions of ER, PR, and HER2 status were previously described in detail [25].

### Follow-up

We followed up the patients at least every 3 months during the first year, and every 6 months during the second and the third year; thereafter, we followed up patients once every year until death or December 31, 2020. We followed up patients by the means of phone call, and outpatient visit. The information of follow-up contained statuses of survival (newly diagnosed diseases, metastasis, recurrence, or death), updated contact information, and treatment information. Overall survival (OS) was primary endpoint for this study, defined as the time from diagnosis until the date of death; the second endpoint was progression free survival (PFS), defined as the time from diagnosis to the date of progression or death. We censored the survival status of subjects at the latest interview or December 31, 2020.

### Statistical analysis

We used multivariate logistic regression models to explore the association of reproductive tract infections with the risk of breast cancer were explored using, and odds ratios (OR) and confidence intervals (95%CI) were calculated. We used Cox proportional hazards models to estimate the hazard ratios (HR) and 95%CI for death and progression of breast cancer in association with reproductive tract infections. The following covariates age at diagnosis (≤ 40, 41–60, > 60), status of menopausal (pre-menopausal or post-menopausal), education level (below junior school, senior high school or above), marital status (unmarried, married/cohabiting, divorced/widowed/separated), status of estrogen receptor (negative or positive), human epidermal growth factor receptor 2 status (positive, equivocal or negative), family history of breast cancer or other cancer (Yes/No), and clinical stage (I/II, III/IV) were adjusted in the models.

To explore the joint effects of female reproductive tract infections and lifetime estrogen exposure on risk and prognosis of breast cancer, we stratified patients by different lifetime estrogen exposure characteristics. We used product terms in the Cox regression models to estimate the interactions. All statistical tests were two-tailed, and *P* < 0.05 was considered to be significant. We performed the above statistical analyses using SPSS, version 25.0.

## Results

### Characteristics of population for case control study

Table 1 showed the distributions of demographic characteristics and common risk factors for breast cancer in cases and controls. Due to frequency-matching, the age distribution was similar between cases and controls. Between cases and controls, the distributions of body mass index (BMI), marital status, parity, menarche age, breastfeeding, history of family cancer were not significantly different, whereas menopausal status and education level were not evenly distributed.

**Table 1:** Characteristics of breast cancer cases and controls

Variables	Cases *n* = 1003 (%)	Controls *n* = 1107 (%)	*P*
**Age (years)**			0.751
≤ 40	253 (25.2)	273 (24.7)	
41–60	568 (56.6)	644 (58.2)	
> 60	182 (18.1)	190 (17.2)	
Mean age (SD)	49.64 (11.79)	49.58 (11.50)	
**Education**			** < 0.001**
Junior middle school or below	491 (49.0)	415 (37.5)	
Senior middle school	262 (26.1)	410 (37.0)	
College or above	228 (22.7)	276 (24.9)	
Unknown	22 (2.2)	6 (0.5)	
**Marital status**			0.750
Never married	31 (3.1)	41 (3.7)	
Married/cohabiting	889 (88.6)	979 (88.4)	
Divorced/widowed/separated	76 (7.6)	85 (7.7)	
Unknown	7 (0.7)	2 (0.2)	
**BMI**			0.118
≤ 23.9	619 (61.7)	722 (65.2)	
24.0–27.9	290 (28.9)	297 (26.8)	
≥ 28.0	92 (9.2)	80 (7.2)	
Unknown	2 (0.2)	8 (0.7)	
**Age at menarche (years)**			0.068
≤ 12.0	125 (12.5)	170 (15.4)	
> 12.0	865 (86.2)	927 (83.7)	
Unknown	13 (1.3)	10 (0.9)	
**Menopausal status**			**0.025**
Pre-menopausal	569 (56.7)	575 (51.9)	
Post-menopausal	431 (43.0)	531 (48.0)	
Unknown	3 (0.3)	1 (0.1)	
**Parity**			0.796
0	70 (7.0)	74 (6.7)	
≥ 1	931 (92.8)	1030 (93.0)	
Unknown	2 (0.2)	3 (0.3)	
**Breastfeeding**			0.314
Never	151 (15.1)	194 (17.5)	
Ever	795 (79.3)	901 (81.4)	
Unknown	57 (5.7)	12 (1.1)	
**Breast cancer history**			0.099
No	949 (94.6)	1067 (96.4)	
Yes	37 (3.7)	27 (2.4)	
Unknown	17 (1.7)	13 (1.2)	
**Oral contraceptive use**			0.560
No	871 (86.8)	865 (78.1)	
Yes	73 (7.3)	80 (7.2)	
Unknown	59 (5.9)	162 (14.6)	

### Associations of reproductive tract infections and estrogen exposure with breast cancer risk and the interaction on the risk

As shown in Table 2, reproductive tract infections were inversely associated with breast cancer risk in the multivariate logistic regression (OR = 0.80; 95% CI, 0.65–0.98). For the lifetime estrogen exposure, the women with a greater number of menstrual cycles had an elevated breast cancer risk (OR = 1.74; 95%CI, 1.38–2.20), whereas there were no associations between other estrogen exposure variables and breast cancer risk.

**Table 2:** Associations of reproductive tract infections and estrogen exposure with breast cancer risk

**Variables**	**Cases** ***n* = 1003**	**Controls** ***n* = 1107**	**OR**^a^	**OR**^b^
**Reproductive tract infections**				
No	775 (77.3)	812 (73.4)	1.00 (reference)	1.00 (reference)
Yes	228 (22.7)	295 (26.6)	**0.81 (0.67, 0.99)**	**0.80 (0.65, 0.98)**
**Number of menstrual cycles**				
≤ 323	302 (30.3)	408 (37.1)	1.00 (reference)	1.00 (reference)
> 323	694 (69.7)	691 (62.9)	**1.36 (1.13, 1.63)**	**1.74 (1.38, 2.20)**
**Reproductive time—Duration of parity—Breastfeeding duration (years)**				
≤ 25	356 (35.8)	339 (30.8)	1.00 (reference)	1.00 (reference)
> 25	639 (64.2)	760 (69.2)	**0.80 (0.67, 0.96)**	0.78 (0.60, 1.02)
**Reproductive time—Duration of all pregnancies—Breastfeeding duration (years)**				
≤ 25	357 (35.9)	340 (30.9)	1.00 (reference)	1.00 (reference)
> 25	638 (64.1)	759 (69.1)	**0.80 (0.67, 0.96)**	0.78 (0.59, 1.02)

^a^ Unadjusted

^b^ adjusted for age, menopause status, education, marital status

We further investigated the modification effects of estrogen exposure on the associations between reproductive tract infections and breast cancer risk. As shown in Supplementary Table 1, the strengths of the associations of reproductive tract infections with the risk were similar between the patients with more number of menstrual cycles (> 323) (OR = 0.74; 95% CI, 0.57–0.96) and those with less number of menstrual cycles (≤ 323) (OR = 0.84; 95%CI, 0.59–1.19), and the heterogeneity was not significant.

### Demographic and clinicopathological characteristics and the associations with reproductive tract infections for the patient cohort

As shown in Table 3, the mean age of participants was 48.00 (SD = 10.69) years and more than two-thirds of patients were pre-menopausal (62.9%) at the time of diagnosis. Nearly 80 percent of the patients were diagnosed with early cancer (79.6%).

**Table 3:** Demographic and clinicopathological characteristics and the associations with female reproductive tract infections for patient cohort

**Characteristics**	**Total**	**Reproductive tract infections**		***P***
	***N* = 4264(%)**	**Yes (*n* = 685)**	**No (*n* = 3579)**	
**Age (years)**				**< 0.001**
≤ 40	1023 (24.0)	239 (34.9)	784 (21.9)	
41–60	2687 (63.0)	406 (59.3)	2281 (63.8)	
> 60	552 (13.0)	40 (5.8)	512 (14.3)	
Mean ± SD	48.00 ± 10.69	44.58 ± 9.19	48.65 ± 10.83	**< 0.001**
**Education**				**0.027**
Junior and below	1909 (47.6)	292 (43.7)	1617 (48.4)	
Senior and above	2098 (52.4)	376 (56.3)	1722 (51.6)	
**Marital status**				**0.010**
Unmarried	117 (2.8)	7 (1.0)	110 (3.2)	
Married/cohabiting	3853 (92.7)	636 (94.5)	3217 (92.4)	
Divorced/widowed/separated	185 (4.5)	30 (4.5)	155 (4.5)	
**Age at menarche**				0.388
≤ 12	558 (13.4)	98 (14.5)	460 (13.2)	
> 12	3614 (86.6)	580 (85.5)	3034 (86.8)	
**Menopause**				**< 0.001**
Pre	2607 (62.9)	526 (78.2)	2081 (59.9)	
Post	1538 (37.1)	147 (21.8)	1391 (40.1)	
**Parity**				**0.004 **
≤ 2	2815 (81.2)	450 (85.7)	2365 (80.4)	
> 2	650 (18.8)	75 (14.3)	575 (19.6)	
**Breastfeeding history**				0.388
No	548 (13.6)	84 (12.5)	464 (13.8)	
Yes	3485 (86.4)	588 (87.5)	2897 (86.2)	
**Family history of breast cancer**				**< 0.001**
No	3718 (89.8)	623 (93.5)	3095 (89.1)	
Yes	421 (10.2)	43 (6.5)	378 (10.9)	
**BMI**				**0.010 **
< 18.5	245 (6.0)	39 (5.8)	206 (6.0)	
18.5–23.9	2412 (58.7)	427 (63.8)	1985 (57.7)	
≥ 24.0	1450 (35.3)	203 (30.3)	1247 (36.3)	
**Oral contraceptive use**				0.085
No	1991 (95.5)	294 (93.6)	1697 (95.8)	
Yes	94 (4.5)	20 (6.4)	74 (4.2)	
**ER**				0.269
Positive	3035 (76.2)	508 (77.9)	2527 (75.8)	
Negative	949 (23.8)	144 (22.1)	805 (24.2)	
**PR**				**0.007**
Positive	2668 (67.1)	466 (71.7)	2202 (66.2)	
Negative	1306 (32.9)	184 (28.3)	1122 (33.8)	
**HER2**				**< 0.001**
Positive	837 (21.8)	106 (16.6)	731 (22.8)	
Equivocal	982 (25.6)	134 (21.0)	848 (26.5)	
Negative	2019 (52.6)	397 (62.3)	1622 (50.7)	
**Stage**				0.515
I/II	3135.(79.6)	501 (80.7)	2634 (79.4)	
III/IV	802.(20.4)	120 (19.3)	682 (20.6)	

Bold indicates statistically significant values

Note, the total number may not be the same because of the missing data

A total of 685 women (16%) reported a history of reproductive tract infections. Patients with female reproductive tract infections were more likely to be pre-menopausal, higher education level, married or cohabiting, lower parity, no family history of breast cancer, normal BMI, PR positive, and HER2 negative, while other characteristics were shown no significant associations with the infections (Table 3). Except for age at menarche, breastfeeding history, BMI, and HER2 status, these characteristics were significantly associated with breast cancer prognosis (Supplementary Table 2).

### Associations of female reproductive tract infections and estrogen exposure with breast cancer prognosis

During the period of follow-up (median 48.5 months, Interquartile Range (IQR): 24.3–76.4 months), 897 disease progressions occurred, including 299 death and 598 recurrence or metastasis. Table 4 showed the associations of female reproductive tract infections and interval of lifetime estrogen exposure with breast cancer prognosis. Compared to patients with non-reproductive tract infections, the infected women had a decreased risk of death (HR = 0.61; 95% CI, 0.40–0.94 for OS) and a better breast cancer PFS (HR = 0.84; 95% CI, 0.65–1.09). For the lifetime estrogen exposure, such as the number of menstrual cycles, intervals of reproductive time, we failed to observe significant association with the prognosis of breast cancer.

**Table 4:** Associations of female reproductive tract infections and estrogen exposure with breast cancer prognosis

Variables	Total	OS			PFS		
		**Events (%)**	**HR (95%CI)**^a^	**HR (95%CI)**^b^	**Events (%)**	**HR (95%CI)**^a^	**HR (95%CI)**^b^
**Reproductive tract infections**							
No	3579	262 (7.3)	1.00 (reference)	1.00 (reference)	494 (13.8)	1.00 (reference)	1.00 (reference)
Yes	685	37 (5.4)	**0.59 (0.42,0.83)**	**0.61 (0.40,0.94)**	104 (15.1)	0.85 (0.68,1.05)	0.84 (0.65,1.09)
**Number of menstrual cycles**							
≤ 327	1105	86 (7.8)	1.00 (reference)	1.00 (reference)	177 (16.0)	1.00 (reference)	1.00 (reference)
> 327	2208	164 (7.4)	0.96 (0.74,1.24)	0.82 (0.57, 1,18)	304 (13.8)	0.87 (0.72,1.05)	0.92 (0.70,1.21)
**Reproductive time—Duration of parity—Breastfeeding duration (years)**							
≤ 25	1147	88 (7.7)	1.00 (reference)	1.00 (reference)	184 (16.0)	1.00 (reference)	1.00 (reference)
> 25	2307	170 (7.4)	0.98 (0.76,1.27)	0.78 (0.51,1.17)	318 (13.8)	0.88 (0.74,1.06)	1.02 (0.73,1.42)
**Reproductive time—Duration of all pregnancies—Breastfeeding duration (years)**							
≤ 25	1100	88 (8.0)	1.00 (reference)	1.00 (reference)	179 (16.3)	1.00 (reference)	1.00 (reference)
> 25	2195	163 (7.4)	0.94 (0.73,1.23)	0.70 (0.47,1.06)	309 (14.1)	0.89 (0.74,1.07)	1.01 (0.72,1.41)

^a^ The univariate COX model

^b^ The multivariate COX model, adjusted for age at diagnosis, menopausal, education, marital status, BMI, ER status, HER2 status, family history, clinical stage

### Joint associations of female reproductive tract infections and estrogen exposure with prognosis of breast cancer

We further examined the modification effects of estrogen exposure on the associations of reproductive tract infections with prognosis of breast cancer. Shown in Table 5, reproductive tract infections decreased the risk of disease progression significantly (HR = 0.52; 95% CI, 0.34–0.79) particularly among women with more number of menstrual cycles (> 327), whereas the association (HR = 1.10; 95% CI, 0.73–1.64) was not significant among patients with less number of cycles (≤ 327); the interaction was significant (*P*_interaction_ = 0.015). Furthermore, the strength of association between breast cancer survival and reproductive tract infections was stronger in patients with more number of menstrual cycles (HR = 0.56, 95% CI, 0.24–1.28) than those with less the cycles (HR = 0.74, 95%CI, 0.38–1.43), though the interaction was not significant. Similar results were observed for other estrogen exposure variables (reproductive time, reproductive time—duration of parity, reproductive time—duration of parity—breastfeeding duration).

**Table 5:** Modification effects of estrogen exposure on the associations between reproductive tract infections and prognosis

**Estrogen exposure**	**Reproductive tract infections**	**Total**	**OS**		**PFS**	
			**Events (%)**	**HR (95%CI)**^a^	**Events (%)**	**HR (95%CI)**^a^
**Number of menstrual cycles**						
≤ 327	No	873	86 (7.7)	1.00 (reference)	177 (16.0)	1.00 (reference)
	Yes	232	17 (7.3)	0.74 (0.38,1.43)	45 (19.3)	1.10 (0.73,1.64)
> 327	No	1886	149 (7.9)	1.00 (reference)	267 (14.1)	1.00 (reference)
	Yes	322	15 (4.6)	0.56 (0.24,1.28)	37 (11.4)	**0.52 (0.34,0.79)**
*P* for interaction		0.286			**0.015 **	
**Reproductive time -Duration of parity—Breastfeeding duration (years)**						
≤ 25	No	886	67 (7.5)	1.00 (reference)	131 (14.7)	1.00 (reference)
	Yes	261	21 (8.0)	0.82 (0.44,1.53)	53 (20.3)	1.13 (0.82,1.76)
> 25	No	1966	158 (8.0)	1.00 (reference)	280 (14.2)	1.00 (reference)
	Yes	341	12 (3.5)	**0.42 (0.22,0.80)**	38 (11.1)	**0.56 (0.38,0.84)**
*P* for interaction		0.137			**0.013 **	
**Reproductive time—Duration of all pregnancies—Breastfeeding duration (years)**						
≤ 25	No	846	67 (7.9)	1.00 (reference)	125 (14.7)	1.00 (reference)
	Yes	254	21 (8.2)	0.79 (0.43,1.47)	54 (21.2)	1.21 (0.83,1.76)
> 25	No	1871	151 (8.0)	1.00 (reference)	272 (14.5)	1.00 (reference)
	Yes	324	12 (3.7)	**0.43 (0.22,0.82)**	37 (11.4)	**0.54 (0.36,0.81)**
*P* for interaction		0.165			**0.005 **	

Bold indicates statistically significant values

^a^ The multivariate COX model, adjusted for age at diagnosis, menopausal, education, marital status, BMI, ER status, HER2 status, family history, clinical stage

## Discussion

Epidemiological research on association of reproductive tract infections with development and progression of breast cancer is currently lacking. In the present study, we firstly found that reproductive tract infections were associated with a decreased risk and a better breast cancer prognosis. Furthermore, it was found that the protective effects on the risk and prognosis were stronger in patients with a longer interval of estrogen exposure than patients with the shorter interval.

It is known that there are an inverse association of acute infections and a positive link of chronic infections with cancer development [26–28]. Acute inflammation stimulated secretion of IL-12, IFN-γ, and other cytokines, which could halt cancer progression by inhibiting angiogenesis and induce the destruction of cancer-associated endothelial cells [29]. Tumor infiltrating leukocytes might become non-specifically activated during acute inflammation and simultaneously upregulate cytotoxic properties, then induce the regression of tumor [30]. Animal experiments also supported our results to some extent. For example, the number of mitotic cells the and size of breast tumor were reduced in breast cancer mice infected with Newcastle disease virus [31]; *Shigella* infection mediate depletion of macrophages and cause tumor regression in mice with breast cancer [32]. Moreover, compared to normal cells, tumor cells were more fragile and vulnerable to fever (accompanied by inflammation) with apoptosis [33, 34]. In addition, a high level of estrogen would inhibit the progression of acute inflammation to chronic inflammation [35, 36]. Therefore, our findings, that the history of reproductive tract infections was associated with a decreasing risk and a better prognosis compared with non-infected patients for the women with a higher level of estrogen exposure, were explainable. Practically, timely diagnosis of infections with routine vaginal swabs and treatment with vaginal probiotics would avoid persisting chronic inflammation and improve the prognosis. For young breast cancer patients who need to preserve their fertility, inositol supplementation for improving the quality of oocytes would influence estrogen level and be likely affect the prognosis of infected patients [37–39]. Therefore, more attention should be paid to the application of inositol.

Previous studies have yielded contradictory findings for the associations between reproductive tract infections and cancer development. Reproductive tract infections such as bacterial vaginosis and Chlamydia trachomatis infection were reported to be associated with a decreasing risk of uterine fibroids [9, 10]; the risk of bladder cancer was reduced among female patients with urinary tract infections but increased among male patients [40], which were consistent with our results. In contrast, Liu et al. found that HPV infection was associated with an increased risk of breast cancer [41]; Lin et al. and Stewart et al. found that pelvic inflammatory diseases related to an increasing risk of urinary tract cancer [16] and ovarian cancer [42], respectively. This inconsistency might be explained by the fact that the intervals of lifetime estrogen exposure in these studies were shorter than that of our study; for example, in Stewart’s study, the proportion of women with multiple parity was higher [42]). 

As for the association between infections and cancer prognosis, previous studies found similar results that infection was associated with a better prognosis of other cancers [43–45]. For example, HPV infection was associated with a prolonging survival among esophageal adenocarcinoma patients [44]; the 5-year survival rate for patients who had empyema after lung cancer was higher than noninfected patients (50% vs 18%) [45]; glioblastoma patients without a postoperative infection had a worse overall survival (HR = 2.3; 95% CI, 1.0-5.3) [43]. On the contrary, childhood infections with pertussis was significantly related to an increased death risk of multiple cancers [3]; pre-diagnostic fever of unknown origin increased the death risk of cancer [46]. One possible reason for the inconsistency was that infections caused by different pathogens might affect cancer progression by different mechanisms: exposure to pertussis toxin may provoke a relative increase in cell proliferation [47]. Another reason was that the previous study failed to adjust potential prognosis factors such as education level, menopausal status, family history of cancer, and clinical stage, which might contribute to the discrepancies [46].

There were some limitations in this study. First, the history of reproductive tract infections was self-reported, which inevitably resulted in recall bias. Moreover, owing to the fact that some of the reproductive tract infections were asymptomatic, the infection rate might be underestimated. However, the recall bias and the underestimation occurred equally in both the case group and control group. This non-differential exposure misclassification might bias study estimators towards the null and reduce test power. Second, the hospital-based design might also lead to selection bias. However, the cases and controls were recruited from the same hospital during the same period, and all subjects must have resided in the Guangzhou area from the same catchment area and resemble each other with regard to those selective factors that led to the hospital admission and use of facilities, resulting in that the cases and controls were comparable. Thus, selection bias was minimized. Third, the frequent visits to the gynecologists for reproductive tract infections may increase the detection of early breast cancer, which would cause detection signal bias. However, breast cancer was usually diagnosed by surgeons in Department of Breast Cancer rather than gynecologists in China, suggesting that the bias was limited. Fourth, the women with long life were prone to expose to more oncogenic factors, which might lead to information bias. We performed a sensitivity analysis with the subjects whose age were younger than 65 (Supplementary Table 3); the similar association indicated that the information bias may not change the results fundamentally. Fifth, we did not thoroughly consider the confounding factors, such as autoimmune diseases. Considering that those parameters would rarely happen to the study subjects, the effect on our results would be limited. We only examined the overall situation of reproductive tract infections but didn’t distinguish the specific pathogens. We have now found that reproductive tract infections were associated with the development and progression of breast cancer, and further studies would be necessary to explore the specific pathogens. Finally, we failed to collect the information about socioeconomic status and treatment which was related to prognosis. However, since the socioeconomic status was associated with education level and treatment was determined by clinicopathological characteristics, adjustment of these characteristics in the statistic models would control the confounding effects to a large extent.

## Conclusions

This study firstly examined the associations of pre-diagnosis reproductive tract infections with risk and prognosis of breast cancer. Our findings did suggest that reproductive tract infections were associated with a decreased risk of breast cancer progression, particularly for patients who had a longer interval of lifetime estrogen exposure. It provided a potential predictor of risk and prognosis and a possible therapeutic regimen for breast cancer.

## Supplementary Information

**Additional file 1.**

## Data Availability

The data that support the findings of this study are available from the corresponding author upon reasonable request.

## Abbreviations

ORs: odds ratios
CIs: confidence intervals
HRs: hazard ratios
PFS: progression-free survival
OS: overall survival
ER: estrogen receptor
PR: Progesterone receptor
HER2: Human epidermal growth factor receptor 2
BMI: Body mass index
