# Supplementary material for: Interaction of reproductive tract infections with estrogen exposure on breast cancer risk and prognosis
Source: BMC Womens Health. 2023 May 8;23:238. doi: 10.1186/s12905-023-02383-3 (PMC10165758; doi:10.1186/s12905-023-02383-3)
Supplement: Supplementary file 1 — Additional file 1. [file 12905_2023_2383_MOESM1_ESM.docx]

| **Supplementary Table 1** Stratified association between reproductive tract infections and breast cancer risk by estrogen exposure | | | | |
| --- | --- | --- | --- | --- |
| **Reproductive tract infection** | **Cases**  **n=1003** | **Controls**  **n=1107** | **OR (95%CI)** ^a^ | **OR (95%CI)** ^b^ |
| **Stratified by number of menstrual cycles** | | | |  |
| ≤323 |  |  |  |  |
| No | 219 (72.8) | 293 (71.8) | 1.00 (reference) | 1.00 (reference) |
| Yes | 82 (27.2) | 115 (28.2) | 0.95 (0.68, 1.33) | 0.84 (0.59, 1.19) |
| ＞323 |  |  |  |  |
| No | 549 (79.1) | 512 (74.1) | 1.00 (reference) | 1.00 (reference) |
| Yes | 145 (20.9) | 179 (25.9) | **0.76 (0.59, 0.97)** | **0.74 (0.57, 0.96)** |
| *P* for interaction | |  | 0.272 | 0.443 |
| **Stratified by Reproductive time - Duration of parity - Breastfeeding duration (years)** | | | | |
| ≤25 |  |  |  |  |
| No | 249 (69.9) | 215 (63.4) | 1.00 (reference) | 1.00 (reference) |
| Yes | 107 (30.1) | 124 (36.6) | 0.75 (0.54, 1.02) | 0.77 (0.55, 1.07) |
| ＞25 |  |  |  |  |
| No | 519 (81.2) | 590 (77.6) | 1.00 (reference) | 1.00 (reference) |
| Yes | 120 (18.8) | 170 (22.4) | 0.80 (0.62, 1.04) | 0.81 (0.62, 1.06) |
| *P* for interaction | |  | 0.723 | 0.578 |
| **Stratified by Reproductive time - Duration of all pregnancies - Breastfeeding duration (years)** | | | | |
| ≤25 |  |  |  |  |
| No | 107 (30.1) | 124 (36.6) | 1.00 (reference) | 1.00 (reference) |
| Yes | 249 (69.9) | 215 (63.4) | 0.75 (0.54, 1.02) | 0.78 (0.56, 1.08) |
| ＞25 |  |  |  |  |
| No | 519 (81.2) | 590 (77.6) | 1.00 (reference) | 1.00 (reference) |
| Yes | 120 (18.8) | 170 (22.4) | 0.80 (0.62, 1.04) | 0.81 (0.61, 1.06) |
| *P* for interaction | |  | 0.737 | 0.578 |
| ^a^ Unadjusted  ^b^ adjusted for age, menopause status, education, marital status | | | | |

| **Supplementary Table 2** Univariate COX regression analyses for demographic and clinical characteristics at baseline with breast cancer prognosis | | | | | | |
| --- | --- | --- | --- | --- | --- | --- |
| **Demographic characteristics** | **Total (%)** | **OS** | |  | **PFS** | |
|  |  | **Events**  **(%)** | **HR (95%CI)** ^a^ |  | **Events**  **(%)** | **HR (95%CI)** ^a^ |
| **Age** | | | |  |  | |
| ≤40 | 1023 (24.0) | 59 (5.8) | 1.00 (reference) |  | 162 (15.8) | 1.00 (reference) |
| 41-60 | 2687 (63.0) | 185 (6.9) | 1.22 (0.91, 1.63) |  | 337 (12.5) | **0.81 (0.67, 0.98)** |
| >60 | 552 (13.0) | 55 (10.0) | **1.69 (1.18, 2.45)** |  | 98 (17.8) | 1.10 (0.85, 1.41) |
| **Education level** | | | |  |  | |
| Junior and below | 1909 (47.6) | 160 (8.3) | 1.00 (reference) |  | 284 (14.8) | 1.00 (reference) |
| Senior and above | 2098 (52.4) | 120 (5.7) | **0.67 (0.53, 0.85)** |  | 282 (13.4) | 0.88 (0.75, 1.04) |
| **Marital status** | | | |  |  | |
| Unmarried | 117 (2.8) | 5 (4.3) | 1.00 (reference) |  | 16 (13.7) | 1.00 (reference) |
| Married/cohabiting | 3853 (92.7) | 261 (6.8) | 1.57 (0.65, 3.81) |  | 535 (13.9) | 0.99 (0.60, 1.63) |
| Divorced/widowed/  separated | 185 (4.5) | 26 (14.1) | **2.93 (1.12, 7.65)** |  | 37 (20.0) | 1.29 (0.72, 2.33) |
| **Age at menarche** | | | |  |  | |
| ≤12 | 558 (13.4) | 35 (6.3) | 1.00 (reference) |  | 74 (13.3) | 1.00 (reference) |
| ＞12 | 3614 (86.6) | 255 (7.1) | 1.13 (0.80, 1.61) |  | 512 (14.2) | 1.08 (0.84, 1.38) |
| **Menopause** | | | |  |  | |
| **Pre** | 2607 (62.9) | 154 (5.9) | 1.00 (reference) |  | 343 (13.2) | 1.00 (reference) |
| **Post** | 1538 (36.1) | 140 (9.1) | **1.55 (1.23, 1.95)** |  | 243 (15.8) | **1.23 (1.04, 1.44)** |
| **Parity** | | | |  |  | |
| ≤2 | 2815 (81.2) | 143 (5.0) | 1.00 (reference) |  | 331 (11.7) | 1.00 (reference) |
| ＞2 | 650 (18.8) | 61 (9.3) | **1.86 (1.38, 2.51)** |  | 103 (15.8) | **1.36 (1.09, 1.70)** |
| **Breastfeeding history** | | | |  |  | |
| No | 548 (13.6) | 39 (7.1) | 1.00 (reference) |  | 83 (15.1) | 1.00 (reference) |
| yes | 3485 (86.4) | 250 (7.2) | 1.04 (0.74, 1.47) |  | 489 (14.0) | 0.95 (0.75, 1.20) |
| **Family history of breast cancer** | | | | | | |
| No | 3718 (89.8) | 278 (7.5) | 1.00 (reference) |  | 544 (14.6) | 1.00 (reference) |
| Yes | 421 (10.2) | 9 (2.1) | **0.37 (0.19, 0.73)** |  | 35 (8.3) | 0.78 (0.56, 1.10) |
| **BMI** | | | | | | |
| <18.5 | 245 (6.0) | 15 (6.1) | 1.00 (reference) |  | 35 (14.3) | 1.00 (reference) |
| 18.5-23.9 | 2412 (58.7) | 159 (6.6) | 1.08 (0.64, 1.84) |  | 332 (13.8) | 0.96 (0.68, 1.36) |
| ≥24.0 | 1450 (35.3) | 113 (7.8) | 1.25 (0.73, 2.15) |  | 212 (14.6) | 1.00 (0.70, 1.44) |
| **Oral contraceptive use** |  |  |  |  |  |  |
| NO | 3724 (94.1) | 260 (6.9) | 1.00 (reference) |  | 528 (14.1) | 1.00 (reference) |
| Yes | 235 (5.9) | 24 (10.2) | 1.34 (0.88, 2.03) |  | 36 (15.3) | 0.99 (0.70, 1.38) |
| ^a^ The univariate COX model. | | | | | | |

| **Supplementary Table 2** (continued) | | | | | | | | |
| --- | --- | --- | --- | --- | --- | --- | --- | --- |
| **Demographic characteristics** | **Total (%)** | | **OS** | |  | **PFS** | | |
|  |  |  | **Events**  **(%)** | **HR (95%CI)** ^a^ |  | **Events**  **(%)** | **HR (95%CI)** ^a^ | |
| **ER** | | | | | | | | |
| Negative | | 949 (23.8) | 114 (12.0) | 1.00 (reference) |  | 198 (20.9) | | 1.00 (reference) |
| Positive | | 3035 (76.2) | 165 (5.4) | **0.43 (0.34, 0.55)** |  | 358 (11.8) | | **0.53 (0.45, 0.63)** |
| **PR** | | | | | | | | |
| Negative | | 1306 (32.9) | 140 (10.7) | 1.00 (reference) |  | 251 (29.2) | | 1.00 (reference) |
| Positive | | 2668 (67.1) | 139 (5.2) | **0.44 (0.35, 0.55)** |  | 303 (11.4) | | **0.53 (0.45, 0.63)** |
| **HER2** | | | | |  |  |  | |
| Negative | 2019 (52.6) | | 154 (7.6) | 1.00 (reference) |  | 305 (15.1) | 1.00 (reference) | |
| Positive/equivocal | 1819 (47.4) | | 119 (6.5) | 1.02 (0.80, 1.29) |  | 235 (12.9) | 1.03 (0.87, 1.22) | |
| **Stage** | | | | |  |  | | |
| I/II | 3135 (79.6) | | 117 (3.7) | 1.00 (reference) |  | 291 (9.3) | 1.00(reference) | |
| III/IV | 802 (20.4) | | 143 (17.8) | **5.95 (4.66, 7.61)** |  | 237 (29.6) | **4.05 (3.40, 4.81)** | |
| ^a^ The univariate COX model. | | | | | | | | |

| **Supplementary Table 3** Associations of female reproductive tract infections with breast cancer prognosis for patients whose age were younger than 65 | | | | | | | | |
| --- | --- | --- | --- | --- | --- | --- | --- | --- |
| **Reproductive tract infections** | **Total** | **OS** | | |  | **PFS** | | |
|  |  | **Events (%)** | **HR (95%CI)** ^a^ | **HR (95%CI)** ^b^ |  | **Events (%)** | **HR (95%CI)** ^a^ | **HR (95%CI)** ^b^ |
| No | 3294 | 231 (7.0) | 1.000 (reference) | 1.000 (reference) |  | 444 (13.5) | 1.000 (reference) | 1.000 (reference) |
| Yes | 653 | 35 (5.4) | **0.607 (0.425, 0.866)** | **0.630 (0.399, 0.995)** |  | 100 (15.3) | 0.874 (0.703, 1.088) | 0.915 (0.701, 1.195) |
| ^a^ The univariate COX model.  ^b^ The multivariate COX model, adjusted for age at diagnosis, menopausal, education, marital status, BMI, ER status, HER2 status, family history, clinical stage. | | | | | | | | |
